# Laparoscopic repair of internal hernia in pregnant women after Roux-en-Y gastric bypass surgery: a case report

**DOI:** 10.1093/jscr/rjag031

**Published:** 2026-01-30

**Authors:** Akram E Farran, Rachel K Thomason, Jessica S Koller Gorham

**Affiliations:** Department of General Surgery, Ochsner Health Clinic, New Orleans, 1514 Jefferson Hwy, Jefferson, LA 70121, United States; Department of General Surgery, University of Queensland, Ochsner Clinical School, New Orleans, 1514 Jefferson Hwy, Jefferson, LA 70121, United States; Department of General Surgery, Ochsner Health Clinic, New Orleans, 1514 Jefferson Hwy, Jefferson, LA 70121, United States

**Keywords:** Roux-en-Y gastric bypass, internal hernia, third trimester, pregnancy, minimally invasive surgery

## Abstract

Laparoscopic Roux-en-Y gastric bypass is a common bariatric operation proven to result in significant weight loss. One recognized serious complication is that of an internal hernia (IH), in which the small bowel protrudes through one of two mesenteric defects created by the Roux-en-Y anatomy. In this case report, we present a successful laparoscopic repair of a symptomatic IH in a patient in the third trimester of pregnancy. This case highlights the challenges in diagnosing vague abdominal pain and emesis during pregnancy, and technical considerations for safe laparoscopic access and monitoring throughout minimally invasive surgery in late gestation.

## Introduction

Roux-en-Y gastric bypass is the second-most commonly performed bariatric surgery in the United States, accounting for over 63 000 procedures in 2023 [1]. Laparoscopic Roux-en-Y gastric bypass (LRYGB) has also demonstrated improvement in gastroparesis symptoms in select patients [2]. The evolution towards an antecolic Roux-limb approach in the laparoscopic era has correlated with a decreased incidence of IH compared to retrocolic technique; however, the risk of IH remains an estimated 2% with defect closure, and up to 7% without closure [3]. Pregnancy may increase the risk of symptomatic IH due to increasing intra-abdominal pressure. Common symptoms of pregnancy along with displacement of abdominal organs by the gravid uterus may obscure the diagnosis of IH and lead to delay in management, thus increasing risk of significant maternal and fetal morbidity and mortality [4]. Traditionally, laparoscopy has been avoided in urgent third-trimester abdominal operations due to risk of uterine injury and risk of spontaneous labor with pneumoperitoneum [4]. We report this case of a third trimester pregnant patient with a history of LRYGB who developed a symptomatic, non-obstructing IH, and underwent successful laparoscopic reduction and repair.

## Case report

**Figure 1:**
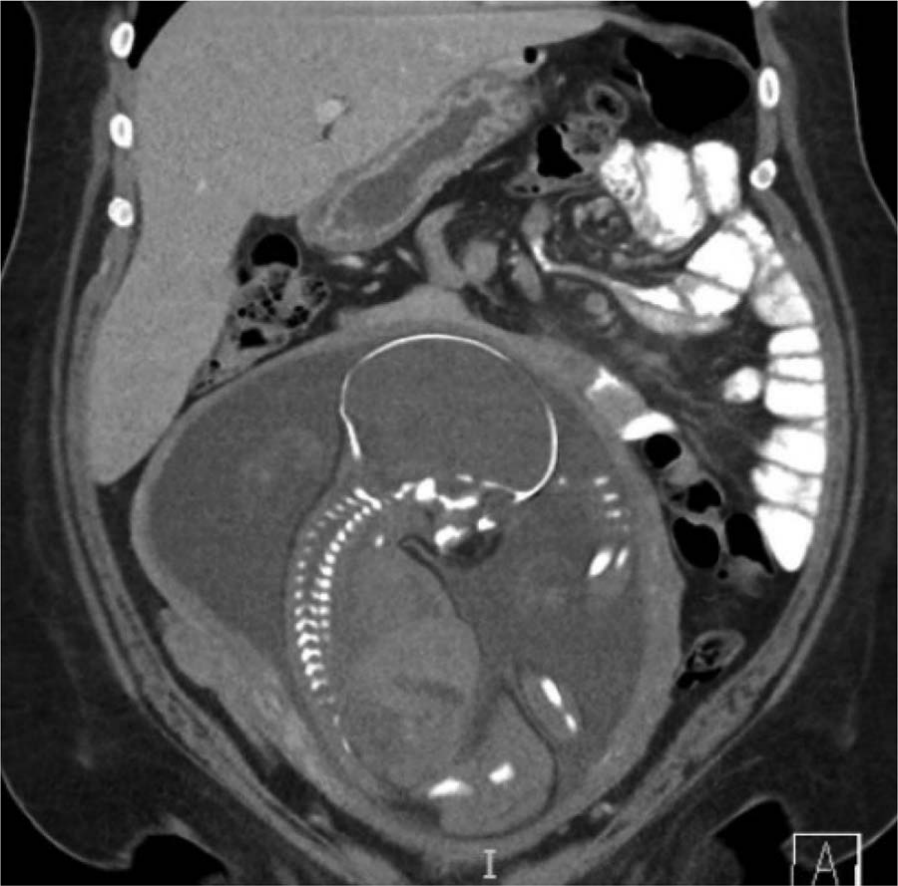
Coronal CT demonstrating left upper quadrant mesenteric swirling with cluster of small intestine entirely on the left abdomen.

**Figure 2:**
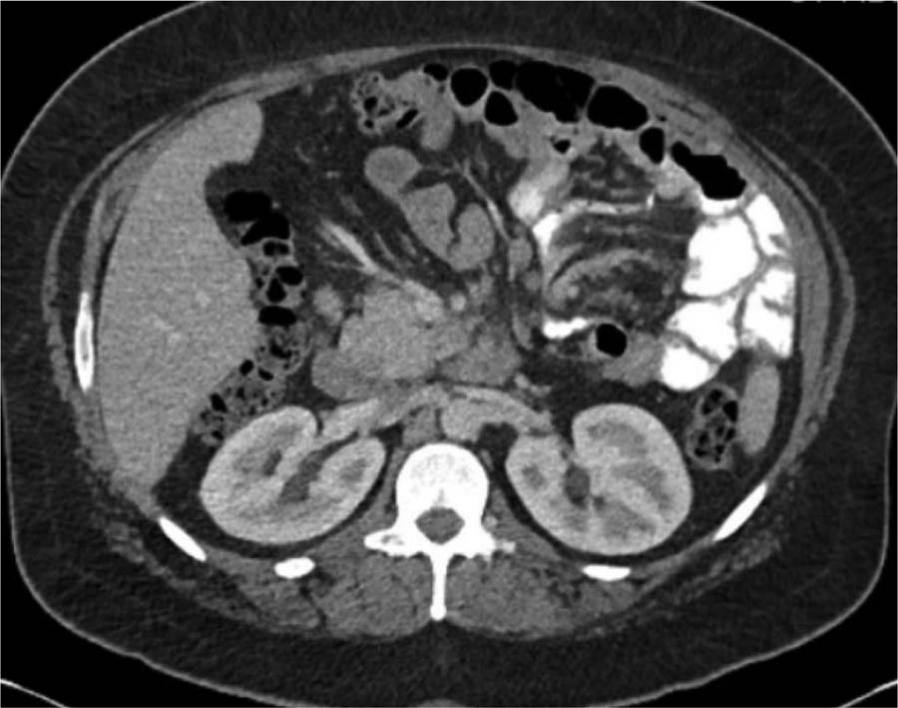
Axial CT demonstrating mesenteric swirling.

A 38-year-old female (Gravida 5, Para 2) at 30 weeks and 1 day gestation with history of a LRYGB performed to treat gastroparesis in the setting of morbid obesity presented to the Emergency Department with acute-onset severe epigastric pain exacerbated by eating. She reported associated nausea and diarrhea, and denied emesis, heartburn, fever, constipation, or dysuria. She was hemodynamically normal with normal fetal heart tones and laboratory results. She underwent esophagogastroduodenoscopy, which revealed a normal-appearing gastric pouch and jejunal mucosa, without evidence of a marginal ulcer. Abdominopelvic CT imaging demonstrated mesenteric swirling with clustering of small intestines entirely on the left abdomen suspicious for IH (Figs 1 and 2). Fluoroscopic small bowel follow through did not demonstrate evidence of small intestinal obstruction. On hospital Day 9, she remained unable to tolerate oral intake, and after multidisciplinary discussion with providers and the patient, she was scheduled for surgical repair.

**Figure 3:**
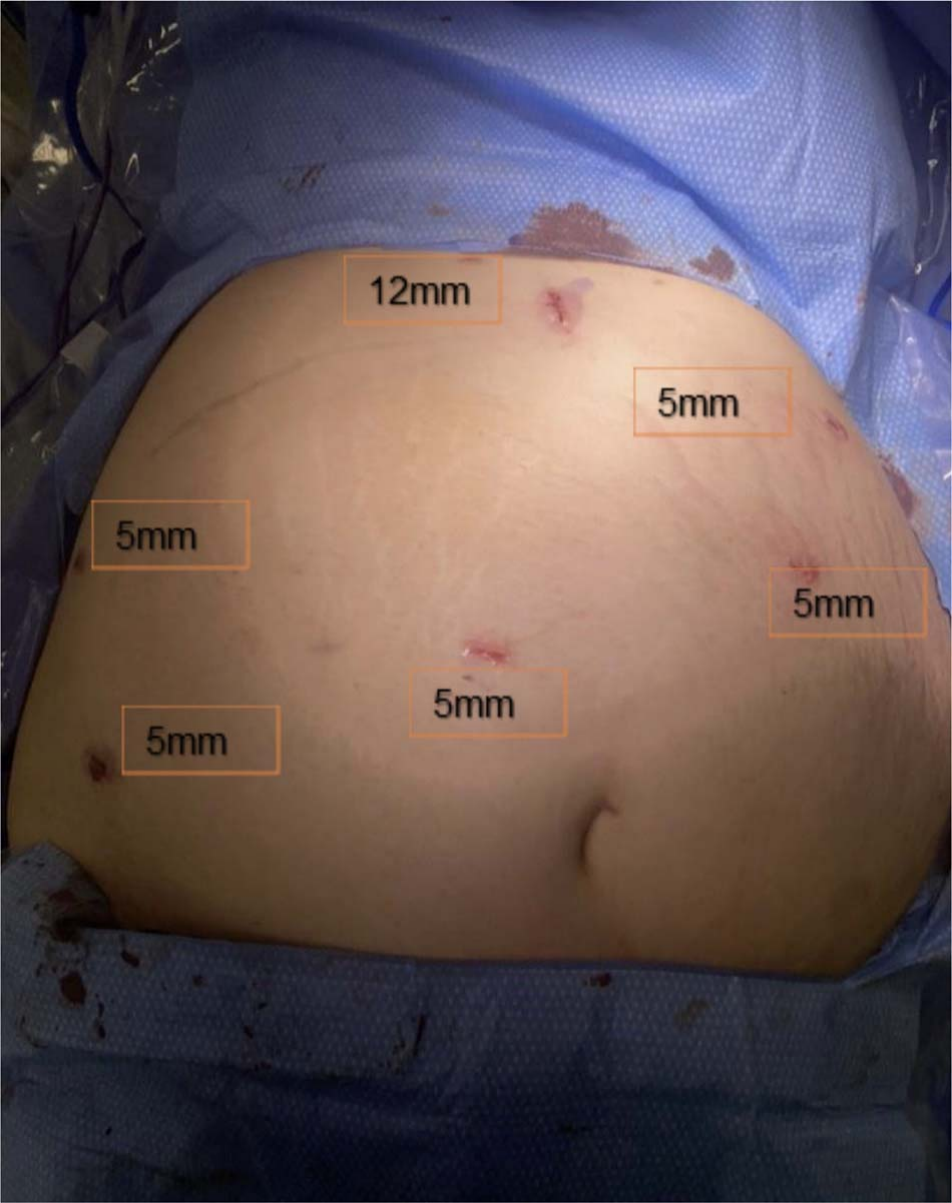
Port placement.

Under OB ultrasound guidance, fundic mapping was performed. The patient was positioned supine on the operating table with 15 degrees elevation on the right to avoid compression of the vena cava. Fetal heart monitoring was performed throughout induction of general anesthesia and intubation. Abdominal access was obtained via open Hasson technique in the upper midline, halfway between the xiphoid and superior uterine border. Following fascial entry, manual palpation of the fundus was performed to confirm safe placement of a 12-mm blunt trocar. The abdomen was insufflated to 15 mmHg. Two right-sided and two left-sided upper lateral 5-mm trocars were placed under direct laparoscopic visualization. A 5-mm trocar was then placed to the right of midline above the fundus to improve visualization of the IH in the setting of her altered anatomy. Trocar placements are shown in Fig. 3. Omental adhesions along the lower abdominal wall were carefully divided with a Ligasure energy device. On initial inspection, all small bowel appeared viable and without dilation. The terminal ileum was identified and the small intestine was sequentially inspected from a distal to proximal approach. While running the bowel, a segment of herniated small intestine appeared to reduce through a mesenteric defect consistent with an IH. Once reduction was complete, a small defect was identified at Petersen’s space and closed with a running 2–0 Ethibond suture. No jejunojejunal mesenteric defect was identified.

One of the challenges of laparoscopy during pregnancy is a lack of constant fetal monitoring. To best navigate this, the CO_2_ insufflation tubing was disconnected and the abdomen fully desufflated every 15 minutes to allow for approximation of the abdominal wall and the uterus. A fetal heart rate monitor was then placed under the sterile drapes and fetal heart tones confirmed. The operation took 105 minutes from incision to closure.

Postoperatively, the patient’s abdominal pain and post-prandial symptoms resolved. She tolerated oral intake on postoperative Day 1 and was discharged on postoperative Day 3. She subsequently delivered a healthy infant via scheduled cesarean section at 37 weeks and 1 day.

## Discussion

Pregnancy after LRYGB has been shown to be safe [5]. However, women remain at risk of an IH or obstruction for months or years after surgery. The most common symptom of an IH is abdominal pain, which could be challenging to discern given the diverse etiology during pregnancy. CT remains the most sensitive diagnostic modality, though concerns regarding fetal radiation exposure may delay imaging. The addition of a small bowel follow-through can greatly increase the diagnostic rate but does not exclude IH in the absence of obstruction [6].

Existing case series and meta-analyses demonstrate a preference for laparotomy in later trimesters because of limited visualization, potential uterine injury during trocar placement, and theoretical associations between pneumoperitoneum and preterm labor [4, 7]. In the largest case series, only 5 of 22 patients underwent laparoscopic repair, and just two were in the third trimester. Out of the 17 patients who underwent laparotomy, there were 3 fetal and 2 maternal deaths. The laparoscopic group had no deaths [7]. In a meta-analysis of 59 cases, 52% were performed via laparotomy [4]. In the absence of comparative studies directly evaluating laparoscopic versus open repair, operative approach is ultimately guided by maternal and fetal stability, bowel viability, gestational age, prior surgical history, and surgeon experience.

Key considerations included continuous intraoperative fetal monitoring and ultrasound-guided fundic mapping to determine safe and tailored trocar placement that accounted for uterine size while maintaining sufficient visualization. As surgeon proficiency in advanced laparoscopy grows, establishing the safety of this approach in late gestation is increasingly important, given its potential benefits over laparotomy of reduced postoperative pain, earlier mobilization, shorter hospital stay, and avoidance of a large midline incision.

## Conclusion

Our case contributes to the limited literature by demonstrating that, in a hemodynamically stable patient without evidence of bowel compromise, laparoscopic repair of an IH can be performed safely in late gestation with appropriate technical modifications. Early recognition and appropriate surgical management is crucial in preventing morbidity and mortality in these cases.

## References

[1] American Society for Metabolic and Bariatric Surgery . Estimate of bariatric surgery numbers. Available from: https://asmbs.org/resources/estimate-of-bariatric-surgery-numbers/.

[2] Masclee GMC, Keszthelyi D, Conchillo JM et al. Systematic review on sleeve gastrectomy or Roux-en-Y gastric bypass surgery for refractory gastroparesis. Surg Obes Relat Dis 2023;19:253–64. 10.1016/j.soard.2022.09.009 36274017

[3] Hajibandeh S, Hajibandeh S, Abdelkarim M et al. Closure versus non-closure of mesenteric defects in laparoscopic Roux-en-Y gastric bypass: a systematic review and meta-analysis. Surg Endosc 2020;34:3306–20. 10.1007/s00464-020-07544-1 32270276

[4] Dave DM, Clarke KO, Manicone JA et al. Internal hernias in pregnant females with Roux-en-Y gastric bypass: a systematic review. Surg Obes Relat Dis 2019;15:1633–40. 10.1016/j.soard.2019.06.009 31378635

[5] Bebber FE, Rizzolli J, Casagrande DS et al. Pregnancy after bariatric surgery: 39 pregnancies follow-up in a multidisciplinary team. Obes Surg 2011;21:1546–51. 10.1007/s11695-010-0263-3 20820939

[6] Ahmed AR, Rickards G, Johnson J et al. Radiological findings in symptomatic internal hernias after laparoscopic gastric bypass. Obes Surg 2009;19:1530–5. 10.1007/s11695-009-9956-x 19756892

[7] Leal-González R, De la Garza-Ramos R, Guajardo-Pérez H et al. Internal hernias in pregnant women with history of gastric bypass surgery: case series and review of literature. Int J Surg Case Rep 2013;4:44–7. 10.1016/j.ijscr.2012.10.006 23108170 PMC3537949

